# Selfie Expectancies Among Adolescents: Construction and Validation of an Instrument to Assess Expectancies Toward Selfies Among Boys and Girls

**DOI:** 10.3389/fpsyg.2018.00839

**Published:** 2018-05-29

**Authors:** Valentina Boursier, Valentina Manna

**Affiliations:** ^1^Department of Humanities, University of Naples Federico II, Naples, Italy; ^2^Association for Social Promotion Roots in Action, Naples, Italy

**Keywords:** selfie, expectancies, adolescents, gender, assessment, measure, validation

## Abstract

Selfie-taking and posting is one of the most popular activities among teenagers, an important part of online self-presentation that is related to identity issues and peer relations. The scholarly literature emphasizes different yet conflicting motivations for selfie-behavior, stressing deeper analysis of psychological factors and the influence of gender and age. Expectancies are “explanatory device[s]” that can help us study adolescent behavior. However, no instruments have been devised that specifically explore the expectations teenagers have about selfies and their influence on selfie-frequency. The current study proposes a short and reliable instrument to identify teen expectancies about selfie-behavior. This instrument was validated using a sample of 646 Italian adolescents (14 to 19 years old) by means of Exploratory Factor Analysis (EFA) and Confirmatory Factor Analysis (CFA). We also explore the relationship between selfie expectancies and selfie-frequency, as well as the role of gender in shaping selfies. Our results point toward a 7-factor model that characterizes expectations toward selfies as a multi-dimensional construct linked to both positive and negative perceptions of the nature and consequences of selfies. The overall model fitted the data sufficiently (χ^2^ = 5067.051, *p* 0.0000; CFI = 0.962; TLI = 0.954; RMSEA ≤ 0.05: 0.035; SRMR = 0.046), showing an adequate reliability of the scale (α = 0.830). Bivariate correlations between selfie expectancies and selfie-frequency (*r* = 0.338, *p* < 0.001) confirmed the convergent validity of the tool. Selfie-sharing is a common practice that is widespread among the participants in this study. Self-promotion represents a positive function of selfies. Selfies promote self-presentation and self-confidence, both in boys and girls. Moreover, selfie expectancies address sexual self-attractiveness, especially among boys. Despite the positive aspects of selfies, our results stress adolescent awareness of the negative consequences of this type of web-exposure. This is especially true among girls, whose selfie-behavior is, paradoxically, more frequent than boys. Self-management through selfie-posting is a positive outcome of selfie-behavior that plays a key role among adolescents, even though the dangers of manipulating selfies in order to garner approval from one’s peers need to be considered. The positive psychometric properties of the measure point toward the need for further research on both generalized and specific selfie-behaviors.

## Introduction

The neologism “selfie” was the Oxford Dictionary’s Word of the Year in 2013 (Oxford Dictionaries, 2013). It commonly refers to a photograph of oneself (alone or with other people) that is taken with a camera, camera phone, or some other hand-held device. Even though the selfie concept addresses several self-portrayal issues (Kiprin, 2013), selfies are typically shared through social media (Sorokowski et al., 2015). Indeed, self-portrayal is one of the most widespread online activities, particularly among adolescents (Lenhart et al., 2010; Kiprin, 2013; Senft and Baym, 2015) and college-age young adults (Katz and Crocker, 2015). According to Lee and Sung (2016), smartphone users take approximately 93 million selfies each day, and approximately 880 billion online photos were shared in 2014. Moreover, 30% of the total photos shared on social networking sites (SNS) in 2014 were selfies posted by adolescents (Locateadoc.com, 2014). It has been estimated that Instagram users alone have shared 238 million photos with the hashtag #selfie, and 128 million photos with the hashtag #me (Weiser, 2015). A recent study in the United States showed that 98% of participants (aged 18 to 24) took selfies, and 69% tended to share selfies 3 to 20 times daily (Katz and Crocker, 2015).

Selfie-taking/sharing certainly represents “one of the dominant forms of content shared in the computer-mediated communication platforms” (Dhir et al., 2016; *p* 0.549). The selfie craze has encouraged greater interest in examining the psychological and psychosocial aspects of this phenomenon, thus feeding the significant debate on both the psychopathological facets of this type of behavior and the growing risks of hyper-pathological conceptualization of common media use (Billieux et al., 2015; Kardefelt-Winther et al., 2017).

According to Nadkarni and Hofmann (2012), social media use fulfills two social needs: self-presentation and the need to belong. Selfie-sharing on SNS improves one’s self-esteem/mood through “likes” (Reich et al., 2018), and seems to be especially related to self-presentation behaviors and relationship construction (Sorokowska et al., 2016; Taylor et al., 2017).

Even though posting selfies allows people to express their own identity and social relationships, other psychological factors might produce different types of selfie behaviors (Albury, 2015). Attitudes toward selfie-taking have been analyzed in three countries by Katz and Crocker (2015). Their study demonstrated the importance of self-presentation and identification in selfie production, as well as the need to receive feedback from one’s peers. Moreover, taking selfies helps people experiment with their appearance, their accessories, and their environment (Kiprin, 2013). Young women declare that selfie-taking helps them to feel authentic (Warfield, 2014). Nguyen and Barbour (2017) recently found that young women consider selfies to be authentic expressions of identity. By contrast, Christoforakos and Diefenbach’s (2016) study found that selfies are associated with a lack of authenticity. They also concluded that young men and women identified both positive aspects (e.g. independence, memory/documentation, relatedness, and control/self-staging) and negative aspects (e.g., illusion/fake, threat to self-esteem, and negative impression on others, and bad picture quality) of selfies.

Recent studies point toward different/conflicting motivations for selfie-taking. For instance, Sung et al. (2016) have shown that attention seeking, archiving, communication, and entertainment motivates selfie-posting on SNS, while also arguing that narcissism considerably predicts selfie-posting frequency. An Italian study, moreover, suggests that various personality traits can predict dissimilar selfie posting behaviors in adolescents and young adults (Baiocco et al., 2016). Other scholars, however, have suggested that narcissism significantly predicts selfie-posting frequency, especially among women (Fox and Rooney, 2015; Sorokowski et al., 2015; Weiser, 2015, 2018; Lee and Sung, 2016; McCain et al., 2016; Barry et al., 2017). Halpern et al. (2016) similarly suggest that selfies have a self-reinforcement effect - that narcissists frequently take selfies in order to maintain positive views of themselves, which in turn increases their narcissism levels.

Etgar and Amichai-Hamburger (2017) have identified three principal motivations behind selfie-taking: selfie-approval, belonging, and documentation. They also suggest that each motivation can be connected to various personality traits. However, unlike previous studies in this area, they did not find a connection between these motivations and narcissism. This somewhat contradictory finding demonstrates that selfies are a multidimensional phenomenon that requires further research. Some research has emphasized the analysis of psychopathological (obsessive) traits among selfie-taking adolescents, oftentimes treating it as a potentially addictive behavior (Balakrishnan and Griffiths, 2017; Griffiths and Balakrishnan, 2018). However, a recent study on the positive psychological effects selfies have on self-presentation strategies has been conducted on young European men and women (Diefenbach and Christoforakos, 2017). The authors’ findings showed that who’s more engaged in selfie-taking considers selfies a good possibility for a selective self-presentation. Strategies associated with self-promotion and/or self-disclosure play an especially important role in supporting various selfie behaviors.

### Age and Gender Differences in Selfie Behavior

Both age and gender influences SNS use, as well as the user’s attitudes and perceptions of Internet-based activities (Dhir et al., 2016). Posting selfies is typically assumed to be a gendered process (Albury, 2015), one that varies according to the type of selfie, selfie frequency, selfie attitudes, and motivations. Males and females tend to use selfies for self-presentation (Katz and Crocker, 2015), however, it has been observed that males and females tend to post different selfies (Sorokowski et al., 2015; Dhir, 2016) and that women are more inclined to post selfies than men (Qiu et al., 2015; Sorokowska et al., 2015, 2016).

Nguyen (2014) has observed that young women (18 to 29 years old) share selfies on Instagram in order to accumulate “likes,” and that the quality of a selfie depends on lighting, scenography, and posture. Nguyen (2014) also found that selfies allow young women to experiment with new and different looks. Recently, Chae (2017) concluded that selfie-editing on social media is related to the average young woman’s attempts to cultivate an ideal form of online self-presentation. Similarly, Nelson (2013) argues that young women share selfies in order to receive positive feedback. For this reason, a selfie code of conduct seems to be especially popular among young women (Warfield, 2014).

Adolescents suggest that selfie-posting could have a negative impact on their self-presentation and social capital (Gibbs et al., 2014). Indeed, they are more likely than adults to engage in a “selfie policy” that emphasizes selecting the ideal photo (Senft and Baym, 2015).

Among young women, selfie posts seem to produce higher self-esteem (Poe, 2015). However, Sorokowska et al. (2016) found that there is no firm relationship between self-esteem and selfie-posting behavior, even though social exhibitionism and extraversion can predict the frequency of selfie-posting among both men and women.

Kim and Chock’s (2015) study states that gender isn’t a significant predictor of selfie behaviors, but it does moderate the relationship between the need for popularity and posting selfies. Indeed, they found that the need for popularity significantly predicts selfie behavior among men, but not women. Meanwhile, Weiser (2015) observes that selfie-posting among women shows a stronger association with leadership and/or authority, while men’s use of selfies seems to be linked primarily to ideas on entitlement and exploitation.

Unfortunately, the scholarly literature on selfies has tended to focus on one gender (Nelson, 2013; Nguyen, 2014; Warfield, 2014), thereby increasing the need to examine selfie behavior among mixed-sex and mixed-age groups (Albury, 2015). Dhir’s (2016) work is one of the few studies to analyze age and gender differences in selfie production and posting. His findings suggest that exploring and building one’s online identity plays a key role in shaping the selfie behavior of both adolescents and young adults. Females and adolescents were found to be more active than males and adults in terms of selfie-taking and posting, collecting photos, and photo-editing. However, male adolescents tend to be influenced by photo-tagging gratifications more than girls, oftentimes using this part of the SNS experience to gain popularity, likes, and comments. Overall, photo-tagging activities tend to satisfy the adolescent’s need for self-construction, identity development, and peer approval (Dhir and Torsheim, 2016).

Young adults seem to have little concern about the risks and consequences of selfie-taking/posting (Katz and Crocker, 2015). Young men and women seem to be conscious of their own privacy, as they tend to be aware that not all selfies should be shared with the general public. People might share their own private images without fully realizing it, which suggests that it is necessary to discriminate between private/personal and public/communicative selfies (Albury, 2015). Moreover, boys seem to have more freedom to exhibit their bodies without risk of disapproval. By contrast, young women’s pictures (and bodies) are subject to a specific kind of surveillance and criticism (Burns, 2014; Albury, 2015). This suggests that culture and gender needs to be evaluated when considering various aspects of selfie behavior (Doring et al., 2016). Furthermore, gender differences often shape the self-presentation strategies of teens who regularly post selfies.

### Expectancies of Internet-Related Behaviors

Expectancies are conscious or unconscious beliefs or thoughts (Goldman, 1994) that reflect the personal beliefs or perceptions about the effect or consequences of a certain behavior (Jung, 2010). The scholarly literature on this topic suggests that personal expectancies influence decisions and behaviors by estimating the consequences of, say, drinking alcohol or engaging in various sexual activities (Dermen and Cooper, 1994; Reich et al., 2010). Indeed, positive outcome expectancies often address and reinforce people’s behavior (Patrick and Maggs, 2009).

Addiction research often sees expectancies as “explanatory device[s]” that can analyze the various decision-making processes that often characterize many addictive behaviors (Reich et al., 2010). Debates on Internet addiction have focused on how estimating positive and negative outcomes can impact one’s behavior. The influence of expectancies on SNS use has been analyzed in young adults (Turel and Serenko, 2012). Dir et al. (2013) have similarly introduced a measure for sexting expectancies and tested its validity on the development of sexting behaviors among undergraduate students. Finally, Brand et al. (2014) have examined the mediating role of cognitive expectations for Internet use and coping styles in the growth and reinforcement of a Generalized Internet Addiction (GIA). By assuming that addictive Internet use is influenced by Internet-related cognitions (Turel et al., 2011; Xu et al., 2012; Lee et al., 2014), several scholars have stressed that Internet-related expectancies play a significant role in the development of GIA in young adults, males and females alike (Brand et al., 2014). In other words, expectancies mediate between specific personality characteristics and the development of Internet addiction. Indeed, the predictive role of expectancies associated with frequent Internet use on various Internet communication disorders has been confirmed in young adults (Wegmann and Brand, 2016; Wegmann et al., 2017). However, no specific gender differences have been analyzed in this area.

### The Present Study

Despite the popularity of selfies among adolescents, there are few instruments and studies that specifically explore teenage beliefs and expectations about selfies and their consequences. We are unaware of any studies that look at how selfie expectancies and gender guide the selfie-behavior of teenagers. Thus, little is known about the quality of the selfie experience among adolescents. Very little information is available about what boys and girls expect from selfies, and the potential correlations between these expectancies and selfie frequency.

The current study aims to validate a reliable instrument that can identify teenage expectancies about selfie production. This involves:

– evaluating the psychometric properties of a selfie expectancies measure;
– exploring the connections between selfie expectancies and how often individuals create selfies;
– analyzing differences that emerge due to gender.

According to the expectancy theory perspective introduced by Dir et al. (2013) and Brand et al. (2014), we assumed that expectancies regarding the consequences of selfies influences selfie practice, which in turn influences future expectancies.

## Materials and Methods

### Participants

According to Gudmundsson (2009), an instrument must be administered to a fairly large sample to be accurately adapted. Brown (2006) and Kline (2011) suggest using at least 10 subjects per item in order to obtain an adequate sample size for Exploratory Factor Analysis (EFA) and Confirmatory Factor Analysis (CFA). Following these suggestions, our convenience sample was composed of 646 adolescents aged 14–19 years (*M* = 16 years; *SD* = 2.519), all of whom were recruited in six secondary schools (I and II grade) from culturally diverse areas of Naples in Southern Italy. The sample was 58.5% male and 41.5% female, and 97.8% of participants have a smartphone. Of this total, 91.4% use it to make phone calls; 94.8% use it to send messages; 81% use it to exchange photos/videos; and 93.5% use it to surf the Internet. Facebook (77%) and WhatsApp (80%) are the two most popular sites for exchanging messages and photos/videos.

All participants were Caucasians from Italian families. All of them participated in this study on a voluntary basis and were informed about the confidentiality/anonymity of the data. There were no incentives for participation and ethical guidelines from the Helsinki Declaration were followed. In accordance with ethical guidelines that are used by the Italian Psychologists Association and the National Psychologists Council, we asked for consent from both the parents of the participants and the relevant school boards. Individual consent was considered when the students voluntarily completed the questionnaire. The Local Ethical Committee approved the study.

### Measures

Participants answered to a self-report anonymous questionnaire during the school hours. It was comprised of four sections: (1) socio-demographic information, (2) mobile phone/social networks/app usage patterns, (3) the Selfie Frequency Scale (SFS), and (4) a newly developed scale to assess selfie expectancies. Four socio-demographic categories were used: gender, age, school year, and school location (town borough).

Within the second section we asked the participants to refer (1) if they have a smartphone; (2) purpose of using smartphones (for calling, to send messages, share photos/videos, surf the Internet); and (3) which apps and social networks they prefer to use for sharing messages and photos/videos.

The *Selfie Frequency Scale* (SFS) (Manna and Boursier, 2017) is an original 19-item tool that was developed to quantify how often adolescents share selfies (α = 0.880). Its structure and dimensions were obtained through a factorial analysis. The measure is based on the assumption that frequency (i.e., the number of times an event occurs) plays a crucial role in determining how adolescents approach the production of selfies. Frequency may provide a consistent measure of problematic selfie behavior from a quantitative point of view. Indeed, frequency may be an indicator of excessive engagement, thus revealing risky behavior. The SFS is a 5-point Likert scale, ranging from 1 (never) to 5 (always), under the query “how often do you…” (e.g., take selfies alone, with a friend, etc.; see **Table 1**). The Selfie Frequency Scale includes three items that refer to both the type and frequency of selfies:

**Table 1:** Selfie Frequency Scale.

***F1 – Standard Selfie*** *(How often do you…)*
1. Take Selfies
2. Take Selfies alone
3. Take Selfies with members of your family
4. Take Selfies with your boyfriend/girlfriend
5. Take Selfies in daily situations (e.g. schools, home…)
6. Take care of your look (hairs, makeup, dresses) when you take Selfies
7. Share Selfies on Facebook or other SNS
8. Share Selfies via smartphone or apps
***F2 – Sexual Selfie***
9. Take Transgressive Selfies
10. Take Selfies with sexually provocative attitudes alone
11. Take Selfies with sexually provocative attitudes with a friend
12. Take Selfies with sexually provocative attitudes with a group of friends
13. Take Selfies with sexually provocative attitudes with family members
14. Take Selfies with sexually provocative attitudes with your boyfriend/girlfriend
15. Take Selfies showing your body (or part of it) naked/almost naked
***F3 – Friendship Selfie***
16. Take Funny Selfies
17. Take Selfies with a friend
18. Take Selfies with a group of friends
19. Take Selfies during particular situations (e.g. parties, events, celebrations…)
**Cronbach’s α:.880**

– Standard Selfie (F1), which includes eight items regarding the “ordinary” nature of the practice, done in everyday situations with familiar people (αF1 = 0.838);
– Sexual Selfie (F2), which is made up of seven items that refer to the tendency to create selfies that feature provocative or sexualized content, alone or with others (αF2 = 0.839);
– Friendship Selfie (F3), which includes four items that address companionship and amusement selfies, shared with others and in specific situations (αF3 = 0.833).

In the newly developed *Selfie Expectancies Scale* (SES) – the first version of which consisted of 54 items – participants had to state “how much selfie-taking…?” by using a 5-point Likert scale. Subsequent statements referred to perceptions about selfies and their possible effects. The scale was developed to both fill a void in the scholarly literature and reinforce the importance of adopting a non-addictive perspective. This measure was based on:

– the various selfie-related behaviors that have been previously addressed in the scholarly literature, with special reference to existing qualitative and quantitative data about selfie outcomes and reasons for taking selfies;
– existing expectancies measures that were developed in previous studies, most notably sexting expectancies (Weisskirch and Delevi, 2011; Dir et al., 2013) and internet use expectancies (Brand et al., 2014);
– focus groups – carried out in various high schools – that featured adolescents who regularly take and post selfies.

Three core points emerged in the focus groups:

– *Worries*: the perceived effects (i.e., risks and benefits) of selfie production on reputation, relationships, etc.;
– *Attitudes*: treating selfies as a way to satisfy various needs, including sexual fantasies, projecting confidence, etc.;
– *Feelings*: how selfies make someone feel (e.g., excited, anxious, guilty, stupid, dirty, sexy, confident, etc.).

We hypothesized that there would be two overarching types of selfie expectancies:

– *positive expectancies*, which encompasses both positive feelings associated with selfie taking and expectations of positive individual/relational behaviors;
– *negative expectancie*s, which entails negative feelings or outcomes that could result from selfie production.

We introduced items referring to negative, positive, and neutral domains. We did not hypothesize, *a priori*, the number of dimensions associated with our expectancies, all of which were based on a factorial analysis. Examples of positive expectancies are included in items stating that selfies might feel participants more popular, more self-confident, or more desired. On the other hand, negative expectancies are expressed under items like “selfie might ruin your relationship/damage your reputation/cause you problems in the future”. Finally, the neutral domain of selfie expectancies is covered by items referring to the widespread use of selfies (e.g., selfie perceived as a habit or a part of current relationships).

### Data Analysis

In order to test the construct validity of the measure, we adopted a random split sample method that divided the overall sample in half. We conducted an EFA on the first half-sample, and then a CFA was performed on the second half-sample in order to confirm the findings from the EFA. This procedure has been adopted in studies that similarly attempted to validate measures for analyzing attitudes (Judd et al., 2014; Martínez et al., 2017). First, we explored the structure of the SES by means of EFA using the software Mplus 6.11 (Muthén and Muthén, 2010). A Robust Maximum Likehood with oblique Geomin rotation was employed because the sample showed a non-normal distribution. Criteria for identifying the factorial solutions were: (1) a factorial saturation of at least 0.30, (2) the analysis of residuals, and (3) the attempt to avoid elevated cross-loadings (Fabrigar et al., 1999). The scree-plot analysis, the Bartlett’s test of sphericity, and KMO measure of sampling adequacy supported the factorial solution. A Confirmatory Factor Analysis (CFA) with Robust Maximum Likehood was employed to verify the identified factorial solution of the SES and its dimensionality. CFI, RMSEA (90% CI), TLI, and SRMR were used as indexes to evaluate the model fit to the data. We also carried on a second order CFA to test the presence of a single implicit psychological construct and to supplementary verify the construct validity. Cronbach’s α, item–total correlations, and factor correlations were adopted to calculate the internal reliability and to examine the internal coherence of the subscales. Bivariate correlations between SES and SFS were conducted to assess the convergent validity and with the purpose of examining the mutual influence of the two measures. A one-sample *t*-test (*t*; *p* < 0.005) was calculated with mean values to compare motivations and draw conclusions about the strongest/less strong reasons to selfie practice. The test value referred to the mean of all motivations on the whole sample. Finally, we evaluated the role of gender by means of one-way ANOVAs (F; *p* < 0.005).

## Results

### Exploratory and Confirmatory Factor Analysis

**FIGURE 1:**
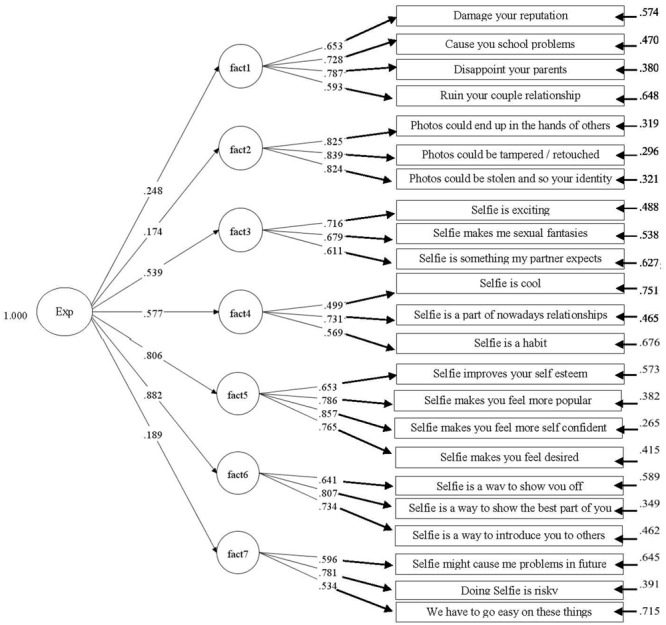
Full model.

During the exploratory analysis, 31 items were removed because of low saturation or high cross-loading. As a result, the final version of the SES consisted of 23 items. EFA on these items yielded all factor loadings greater than 0.3. Both the scree-plot and the eigenvalue suggested a 7-factor solution which explains the 51.26% of variance (Bartlett’s test of the sphericity: 0.828) (**Table 2**). The solution was then verified by means of CFA. The overall model fitted the data adequately (χ^2^ = 5067.051 *p* = 0.0000; CFI = 0.962; TLI = 0.954; RMSEA < = 00.05: 0.035; SRMR = 0.046) (**Figure 1**).

**Table 2:** Results from Exploratory Factor Analysis.

	*F1*	*F2*	*F3*	*F4*	*F5*	*F6*	*F7*
***F1 – Relational Worries*** *(How much selfie-taking…)*					
1. Might damage your reputation	**0.745**	–0.028	0.081	0.003	–0.049	–0.009	0.0135
2. Might cause you school problems	**0.707**	0.010	–0.003	–0.056	–0.025	0.114	0.037
3. Might disappoint your parents	**0.776**	0.054	–0.059	0.007	0.003	0.042	–0.046
4. Might ruin your romantic relationship or the chance to have a relation with someone you are in	**0.573**	0.067	–0.099	0.093	0.005	–0.236	–0.173
***F2 – Web-related anxieties***					
5. Might worry you because your photos could end up in the hands of other people who could use them in an unapproved manner	0.172	**0.745**	0.049	–0.064	0.002	–0.017	–0.002
6. Might worry you because your photos could be retouched	0.024	**0.825**	0.002	–0.092	–0.018	0.012	–0.086
7. Might worry you because your photos/identity could be stolen	–0.101	**0.888**	–0.020	0.088	–0.009	0.007	0.078
***F3 – Sexual desire***					
8. Is exciting	0.064	–0.006	**0.560**	0.029	0.215	0.070	0.004
9. Improves your sexual fantasies	0.011	–0.009	**0.902**	0.001	–0.008	–0.061	–0.014
10. Is something your partner expects/would expect from you	–0.116	0.082	**0.576**	–0.001	–0.093	0.135	–0.030
***F4 – Ordinary practice***.					
11. Is cool	0.034	0.110	–0.017	**0.416**	0.121	0.109	–0.023
12. Is a part of current relationships	–0.075	0.033	0.108	**0.526**	0.021	0.182	0.013
13. Is a habit	0.028	–0.070	–0.024	**0.725**	–0.048	–0.039	0.019
***F5 – Self-confidence***					
14. Improves your self-esteem	0.014	0.034	0.007	0.137	**0.526**	0.161	–0.323
15. Makes you feel more popular	–0.025	–0.050	0.051	–0.008	**0.533**	0.301	0.229
16. Makes you feel more self-confident	–0.020	–0.021	–0.011	0.012	**0.914**	–0.060	–0.027
17. Makes you feel desired	–0.063	–0.022	0.184	–0.018	**0.620**	0.122	0.188
***F6 – Self-presentation***					
18. Is a way to show you off	0.029	0.029	0.045	0.095	–0.088	**0.750**	0.045
19. Is a way to show to the others the best part of you	0.032	–0.036	0.060	0.045	0.208	**0.572**	–0.150
20. Is a way to introduce you to the others	–0.027	0.010	–0.052	0.067	0.279	**0.522**	–0.097
***F7 – Generalized risks***					
21. Might cause you problems in the future	0.240	0.152	–0.187	0.003	0.099	–0.076	**0.384**
22. Is risky	0.251	0.287	–0.016	0.042	0.006	0.033	**0.408**
23. Need to be careful	0.101	0.143	0.058	0.067	0.180	–0.202	**0.417**
**Cronbach’s α**	**0.755**	**0.861**	**0.673**	**0.600**	**0.837**	**0.737**	**0.621**

The emerged structure shows that the various expectancies toward selfies suggest the presence of a multicomponent construct that includes references to several different dimensions: the Self, sexual issues, the relational component of identity, and positive or negative perceptions of selfie-behavior. Seven factors were considered:

• *Relational Worries* (F1): this includes the negative consequences of selfie-behavior. They reflect both the individual and relational characterization of selfie-sharing, placing special emphasis on the effects on the self, the family, and personal relationships.
• *Web-related anxieties* (F2): features items that refer to the negative consequences related to the online nature of selfie-behavior. This factor suggests that selfie sharing is perceived by adolescents as being potentially dangerous.
• *Sexual desire* (F3): this factor reinforces the idea that sexuality is an important component of selfies, oftentimes spurring fantasies and feelings of excitement that can be shared with a partner.
• *Ordinary practice* (F4): this factor focuses on items that emphasize the ordinary (and ubiquitous) nature of selfies among adolescents. This dimension might reduce the adolescent’s ability to identify the risks connected to behavior that is often considered to be “normal.”
• *Self-confidence* (F5): this factor highlights the reinforcing nature of selfies, with special emphasis placed on the extent to which adolescents expect selfies to increase their self-esteem and improve their status among others, thereby increasing their confidence.
• *Self-presentation* (F6): this factor focuses on how adolescents use selfies to show parts of themselves or a specific aspect of the self to the world. The risk of self-manipulation should be considered here, due to a powerful need to be accepted and “liked” by others during this stage of the life cycle.
• *Generalized risks* (F7): this factor keys in on the idea that selfies are dangerous. On the one hand, this factor shows that adolescents understand that there are risks associated with selfie-taking. On the other hand, these risks might be protective in nature, a means of encouraging adolescents to adopt a safer approach to selfie use.

### Convergent Validity

Bivariate correlations showed that Selfie Expectancies and Selfie Frequency assess distinct constructs strongly interrelated (*r* = 0.338; *p* < 0.001), thus confirming the convergent validity of the tool (**Table 3**). *Self-confidence* is strongly correlated with *Selfie frequency* (*r* = 0.413; *p* < 0.001). Moreover, *Self-presentation* correlates most with *Standard selfie* (*r* = 0.415; *p* < 0.001). One of the strongest correlations emerges between *Sexual desire* and *Sexual selfie* (*r* = 0.474; *p* < 0.001).

**Table 3:** Correlations among scales and factors.

	SFS	SFS-F1	SFS-F2	SFS-F3	SES	SES-F1	SES-F2	SES-F3	SES-F4	SES-F5	SES-F6	SES-F7
**Correlations**					
Selfie Frequency Scale	1											
SFS-F1 – *Standard Selfie*	0.927**	1										
SFS-F2 – *Sexual Selfie*	0.603**	0.349**	1									
SFS-F3 – *Friendship Selfie*	0.777**	0.683**	0.218**	1								
Selfie Expectancies Scale	0.338**	0.330**	0.269**	0.158**	1							
SES-F1 – *Relational Worries*	–0.064	–0.035	–0.142**	–0.030	–0.378**	1						
SES-F2 – *Web-related anxieties*	–0.067	–0.089	–0.016**	–0.084*	–0.512**	0.442**	1					
SES-F3 – *Sexual desire*	0.261**	0.157**	0.474**	0.062	0.420**	–0.181**	–0.009	1				
SES-F4 – *Ordinary practice*	0.130**	0.124**	0.069	0.095*	0.450**	0.003	0.009	0.196**	1			
SES-F5 – *Self-confidence*	0.413**	0.261**	0.290**	0.211**	0.582**	–0.162**	–0.107**	0.358**	0.120**	1		
SES-F6 – *Self-presentation*	0.311**	0.415**	0.221**	0.180**	0.558**	–0.145**	–0.051	0.333**	0.168**	0.611**	1	
SES-F7 – *Generalized risks*	–0.111**	–0.143**	–0.033	–0.043	–0.398**	0.329**	0.370**	–0.096*	–0.008	–0.047	–0.114**	1

*^∗∗^The correlation is significant at level 0.01 (2-codes).*

*^∗^The correlation is significant at level 0.05 (2-codes).*

Positive expectancies are most negatively correlated with *Web-related anxieties* (*r* = -0.512; *p* < 0.001), and are most positively correlated with *Self-confidence* (*r* = 0.582; *p* < 0.001) and *Self-presentation* (*r* = 0.558; *p* < 0.001). These last two factors also produce the highest levels of inter-correlation (*r* = 0.611; *p* < 0.001) and have the strongest correlation in terms of both frequency and expectancies about selfies. A strong correlation has also been found among *Relational Worries* and *Web-related anxieties* (*r* = 0.442; *p* < 0.001).

### Reliability

Cronbach’s alphas showed an adequate reliability of the scale (α = 0.830) and an acceptable internal consistency for the subscales (α_F1_ = 0.755; α_F2_ = 0.861; α_F3_ = 0.673; α_F4_ = 0.600; α_F5_ = 0. 837; α_F6_ = 0.737; α_F7_ = 0.621). The solution revealed sufficient inter-item correlations (from 0.255 to 0.742) and significant inter-correlations among its factors (*p* < 0.001).

In terms of the correlations between SES and SFS, *Self-confidence* is strongly correlated with Selfie frequency (*r* = 0.413; *p* < 0.001). Moreover, *Self-presentation* produces the highest correlation with *Standard selfie* (*r* = 0.415; *p* < 0.001). One of the strongest correlations emerges between *Sexual desire* and *Sexual selfie* (*r* = 0.474; *p* < 0.001).

### Descriptives and Results From *t*-Test

Data from the SFS revealed that selfies are a widespread practice: only 3.6% of our sample have never taken a selfie. They are a ubiquitous feature of contemporary youth culture, oftentimes being created during special events (*M* = 3.54; *SD* = 1.109) and in daily situations (*M* = 2.81; *SD* = 1.145). The selfie is a tool for socialization. It is usually taken 2–4 times a week with a boyfriend/girlfriend (84%) or friends (87%), and feature humorous content (64.9%). Selfies are also shared with others by 82% of participants, especially on SNS (59.3%) or WhatsApp groups (60.2%).

Descriptives from the SES and results from the one-sample *t*-test (**Table 4**) reveal that selfies have a reinforcement function. Indeed, our findings show that selfies are used as a tool to manage self-confidence (F5: *M* = 2.45; *SD* = 1.055), increase self-esteem (*M* = 2.42; *SD* = 1.254), make adolescents feel more self-confident (*M* = 2.52; *SD* = 1.298), and desired (*M* = 2.45; *SD* = 1.302). Secondly, we found that selfies were often used as an instrument to present oneself (F6: *M* = 2.40; *SD* = 1.036), allowing our participants to show off (*M* = 2.53; *SD* = 1.229), introduce themselves to others (*M* = 2.46; *SD* = 1.224), and reveal the best part of themselves to others (*M* = 2.22; *SD* = 1.249).

**Table 4:** Descriptives and results from one-sample *t*-test.

	*M*	*SD*	*t (1.966)*	*Sig. (2-tailed)*
***F1 – Relational Worries***	1.65	0.772	–23.255	0.000
Might ruin romantic relationship	1.94	1.207	–8.708	0.000
Might damage reputation	1.74	0.973	–16.171	0.000
Might disappoint parents	1.55	0.968	–20.933	0.000
Might cause school problems	1.36	0.815	–30.923	0.000
***F2 – Web-related anxieties***	2.60	1.279	4.636	0.000
Photos could end up in the hands of other people	2.84	1.440	8.111	0.000
Photos/identity could be stolen	2.57	1.498	3.599	0.000
Photos could be retouched	2.41	1.368	0.803	0.422
***F3 – Sexual desire***	1.64	0.803	–22.661	0.000
Is exciting	1.80	1.014	–13.853	0.000
Improves your sexual fantasies	1.47	0.978	–22.936	0.000
Is something your partner expects/would expect from you	1.65	1.040	–17.152	0.000
***F4 – Ordinary practice***	3.58	0.931	33.147	0.000
Is cool	3.86	1.203	31.530	0.000
Is a habit	3.78	1.161	30.905	0.000
Is a part of current relationships	3.10	1.309	14.303	0.000
***F5 – Self-confidence***	2.45	1.055	2.168	0.031
Increases self-esteem	2.42	1.254	1.210	0.027
Makes feel more self-confident	2.52	1.298	3.166	0.002
Makes feel desired	2.45	1.302	1.773	0.017
Makes popular	2.41	1.257	1.093	0.275
***F6 – Self-presentation***	2.40	1.036	1.004	0.036
Is a way to show you off	2.53	1.229	3.234	0.001
Is a way to introduce you to the others	2.46	1.224	2.124	0.034
Is a way to show to the others the best part of you	2.22	1.249	–2.869	0.004
***F7 – Perceived risks***	2.36	0.893	0.174	0.862
Might cause future problems	1.63	0.950	–19.360	0.000
Need to be careful	3.00	1.265	12.770	0.000
Is risky	2.46	1.208	2.200	0.028

In terms of negative expectancies, our participants appear particularly worried about web-related anxieties (F2: *M* = 2.60; *SD* = 1.279) and their relationship to various identity issues. They seem especially worried that their photos may end up in the hands of other people who could use them in an unapproved manner (*M* = 2.83; *SD* = 1.440); that their own photos/identity could be stolen (*M* = 2.57; *SD* = 1.498); and that their photos could be tampered with or retouched (*M* = 2.41; *SD* = 1.368). Interestingly enough, web-related anxieties tend to overshadow the positive expectancies (F5 and F6) mentioned earlier.

Our participants are less likely to think that selfies are dangerous (F7: *M* = 2.36; *SD* = 0.893), as many of them refuse to believe that future problems could arise from taking selfies (*M* = 1.63; *SD* = 0.950). However, they are more likely to recognize the necessity to be careful with selfies (*M* = 3.00; *SD* = 1.265), considered as a risky practice in general (*M* = 2.46; *SD* = 1.208). In a similar vein, our participants are not especially concerned about the negative consequences selfies might have on one’s self, one’s family, or one’s personal relationships (F1: *M* = 1.65; *SD* = 0.772). Furthermore, they do not think that selfies are capable of ruining romantic relationships (*M* = 1.94; *SD* = 1.207), damaging one’s reputation (*M* = 1.74; *SD* = 0.973), disappointing parents (*M* = 1.55; *SD* = 0.968), or causing school problems (*M* = 1.36; *SD* = 0.815).

Overall, the highest scores were registered in the selfie as an ordinary practice concept (F4: *M* = 3.58; *SD* = 0.931). This suggests that our participants see selfies as a common feature of adolescence – a cool trend (*M* = 3.86; *SD* = 1.203), a habit (*M* = 3.78; *SD* = 1.161) or a key part of contemporary relationships (*M* = 3.10; *SD* = 1.309).

Finally, the sexual aspects of selfies received the lowest scores (F3: *M* = 1.64; *SD* = 0.803). Items from this dimension include: selfies are exciting (*M* = 1.80; *SD* = 1.014); selfies promotes sexual fantasies (*M* = 1.47; *SD* = 0.978); and selfies are something my partner expects/would expect from me (*M* = 1.65; *SD* = 1.040). These results align with the findings from the SFS. Indeed, only 15.9% of participants claimed to have taken transgressive selfies, while only 11.1% claimed to have taken provocative selfies. As a result, it is safe to say that although selfies have a sexual component, adolescents don’t consider this a major feature of the selfie-taking process.

### Gender Differences

Our findings suggest that a moderate role is played by gender. The SFS found that although selfies, in general, are more common among females (*M*_F_ = 3.79; *SD*_F_ = 0.912; *M*_M_ = 3.12; *SD*_M_ = 0.959), selfies with sexual content are more common among males (*M*_F_ = 1.21; *SD*_F_ = 0.628; *M*_M_ = 1.35; *SD*_M_ = 0.778). Indeed, males registered a higher prevalence on all items related to the sexual, provocative, and transgressive nature of selfies. No gender differences were found in items that focused on friends, SNS use, and apps, thus confirming that selfies are used primarily as a tool for managing and sharing information about relationships.

Nonetheless, some gender differences were found in several factors. ANOVAs performed on the SES, for instance, revealed significant preoccupation levels among girls. As shown in **Table 5**, girls report more web-related anxieties (F2: *M*_F_ = 2.86; *SD*_F_ = 1.337; M_M_ = 2.40; *SD*_M_ = 1.201) and perceived risks (F7: M_F_ = 2.46; *SD*_F_ = 0.911; M_M_ = 2.30; *SD*_M_ = 0.875). The only concern that is greater among males than among females is the fear that selfies might ruin a personal relationship (M_F_ = 1.73; *SD*_F_ = 1.095; M_M_ = 2.09; *SD*_M_ = 1.261).

**Table 5:** One-way ANOVAs by gender with means and standard deviations for gender variables.

	*M* (*SD*)		F	Sig.	η^2^
	*Males*	*Females*			
***F1 - Relational Worries***	1.67 (0.732)	1.61 (0.824)	1.132	0.288	0.002
Might ruin romantic relationship	2.09 (1.26)	1.73 (1.09)	14.026	0.000*	0.132
Might damage reputation	1.74 (0.973)	1.76 (1.04)	0.246	0.620	0.000
Might disappoint parents	1.72 (0.920)	1.56 (1.06)	0.003	0.959	0.000
Might cause school problems	1.55 (0.896)	1.34 (0.741)	0.739	0.390	0.001
***F2 – Web-related anxieties***	2.40 (1.20)	2.86 (1.33)	20.622	0.000*	0.132
Photos could end up in the hands of other people	2.63 (1.39)	3.10 (1.45)	16.617	0.000*	0.126
Photos/identity could be stolen	2.36 (1.43)	2.88 (1.53)	18.752	0.000*	0.129
Photos could be retouched	2.23 (1.29)	2.65 (1.43)	14.515	0.000*	0.133
***F3 – Sexual desire***	1.83 (0.890)	1.36 (0.559)	56.248	0.000*	0.181
Is exciting	1.99 (1.09)	1.53 (0.813)	33.583	0.000*	0.150
Improves your sexual fantasies	1.69 (1.11)	1.16 (0.632)	49.846	0.000*	0.173
Is something your partner expects/would expect from you	1.82 (1.11)	1.42 (0.871)	24.108	0.000*	0.012
***F4 – Ordinary practice***	3.55 (0.944)	3.61 (0.913)	0.467	0.494	0.001
Is cool	3.82 (1.21)	3.92 (1.19)	0.995	0.319	0.002
Is a habit	3.69 (1.21)	3.91 (1.06)	5.727	0.017	0.009
Is a part of current relationships	3.17 (1.29)	3.01 (1.33)	2.368	0.124	0.004
***F5 – Self-confidence***	2.48 (1.06)	2.39 (1.04)	1.098	0.295	0.002
Improves self-esteem	2.33 (1.21)	2.55 (1.29)	4.685	0.031	0.007
Makes feel more self-confident	2.50 (1.30)	2.56 (1.28)	0.383	0.536	0.001
Makes feel desired	2.60 (1.32)	2.24 (1.23)	11.798	0.001*	0.018
Makes popular	2.53 (1.28)	2.25 (1.19)	8.025	0.005*	0.012
***F6 – Self-presentation***	2.47 (1.05)	2.29 (1.00)	5.070	0.025	0.008
Is a way to show you off	2.68 (1.30)	2.31 (1.25)	13.231	0.000*	0.021
Is a way to introduce you to the others	2.53 (1.24)	2.36 (1.16)	3.223	0.073	0.005
Is a way to show to the others the best part of you	2.23 (1.25)	2.20 (1.24)	0.091	0.763	0.000
***F7 – Perceived risks***	2.29 (0.875)	2.46 (0.910)	5.341	0.021	0.008
Might cause future problems	1.63 (0.950)	1.63 (0.952)	0.007	0.934	0.000
Need to be careful	2.96 (1.24)	3.05 (1.29)	0.749	0.387	0.001
Is risky	2.30 (1.18)	2.70 (1.20)	17.696	0.000*	0.027

*^∗^*p < 0.005*.*

Boys are more likely to see selfies in a sexual light, placing special emphasis on self-attractiveness (F3: M_F_ = 1.37; *SD*_F_ = 0.559; M_M_ = 1.83; *SD*_M_ = 0.890). Selfies are exciting to boys; they contribute to their sexual fantasies and often lead to expectations that their partners should create similarly explicit content. Boys also have greater positive expectancies, as they tend to consider selfies as self-presentation tools (F6: M_F_ = 2.29; *SD*_F_ = 1.006; M_M_ = 2.47; *SD*_M_ = 1.051) that are connected to their sexual desires.

Since girls are more likely to regard selfie-taking as a risky practice (M_F_ = 2.70; *SD*_F_ = 1.209; M_M_ = 2.30; *SD*_M_ = 1.188), they might be more cognizant of the negative consequences of posting selfies. Among boys, by contrast, selfies are tied to excitement, sexual desire, and managing their self-image. Selfies, in short, help boys feel more desired (*M*_F_ = 2.24; *SD*_F_ = 1.237; *M*_M_ = 2.60; *SD*_M_ = 1.328), providing them with a venue in which they can show off to their friends (*M*_F_ = 2.31; *SD*_F_ = 1.256; *M*_M_ = 2.68; *SD*_M_ = 1.308).

These findings should consider the magnitude of effect size, as given by the η^2^. According to Pierce et al. (2004), a η^2^ value lower than 0.13 is considered small, a value from 0.13 to 0.23 is moderate, and values higher than 0.23 are considered large. Using this criterion as a guide, our data set revealed moderate effects of gender on Sexual desire and Web-related anxieties. In fact, 18.1% of the variance found in the Sexual desire dimension can be attributed to gender, especially items pertaining to excitement (η^2^ = 0.150) and sexual fantasies (η^2^ = 0.173). Moreover, 13.2% of the variance in Web-related anxieties is due to gender, as a moderate effect has been found in all of the items (selfie practice may ruin a personal relationship: η^2^ = 0.132; photos could end up in the hands of other people: η^2^ = 0.126; photos could be tampered with or retouched: η^2^ = 0.133; and photos/identity could be stolen: η^2^ = 0.129). All the other differences that arose due to gender are significant, but not to the same extent as the items discussed above. Nonetheless, the idea that boys are more involved in the sexualized aspects of selfie-behavior, and that girls are more worried about the negative consequences of selfies, requires further research.

## Discussion

Unfortunately, the scholarly literature that has emerged in recent years on selfie culture doesn’t address age and gender differences. Scholars have shown that both age and gender affect the way the Internet and SNSs are utilized (Albury, 2015), and yet few studies have investigated social media use and selfie practices among people of different age and gender (Dhir et al., 2016, 2017).

This study contributes to the ongoing scientific debate on the psychological functions and attitudes implied in selfie-behavior, as well as the motivations behind this practice. Moreover, the trend to medicalize everyday behavior has influenced this study by allowing us explore selfie production among adolescents without adopting an addiction/medicalized perspective (Starcevic et al., 2018).

Furthermore, this study has a unique age/gender viewpoint. Indeed, these themes were explored with special reference to selfie diffusion among adolescents, many of whom are engaged in self-definition, identity construction, and relational interactions. In fact, selfies may help individuals express and fortify their own identity in an online context. According to some scholars (Nadkarni and Hofmann, 2012; Nguyen, 2014; Katz and Crocker, 2015; Sorokowska et al., 2016; Diefenbach and Christoforakos, 2017; Etgar and Amichai-Hamburger, 2017; Taylor et al., 2017; Reich et al., 2018), self-presentation, self-promotion, and self-approval are prominent features of selfie experience.

If we assume that expectations play a key role in determining people’s behavior, then it is safe to say that a measure that is specifically oriented to assess selfie expectations could be especially valuable to both scholars and practitioners. This study aimed to validate a psychometric tool that can be used to assess expectations toward selfies among adolescents. This tool overcomes the shortcomings of extant instruments, and allows us to better recognize what motivates adolescents to create selfies, without necessarily treating it as symptomatic behavior or a unique psychiatric issue.

The proposed 7-factor model fitted the data adequately, while also highlighting that positive, negative, and neutral consequences need to be considered. Our sample showed that selfies were most often created via smartphones, and that selfies are a key component of contemporary adolescence. Selfie creation is neither positive nor negative, but strongly related to the customs and habits of millennials.

Positive expectations toward selfies are related to the idea that selfies are a tool for self-presentation and self-promotion, which in turn are related to self-disclosure and self-management strategies. The use of selfies to garner approval (and feelings of gratification) from one’s peers and improve one’s self-esteem, self-confidence, and popularity has been confirmed by previous research in this area (Etgar and Amichai-Hamburger, 2017). According to Diefenbach and Christoforakos (2017), selfie-taking may play a key role in self-presentation and self-promotion. Moreover, our study found that the process of taking selfies among adolescents often focuses on choosing what to show others, which suggests that adolescents fear having their images tampered with or manipulated (McLean et al., 2015; Chae, 2017). Additionally, the sexual aspects of selfies emerged as a constitutive dimension of selfie expectations, especially among boys who were concerned with self-attractiveness issues. In other words, selfies are often used by our participants to manage a host of identity-related issues.

Differently from Diefenbach and Christoforakos’ (2017) study on young adults, neither positive aspects due to the authentic expression of oneself, nor concerns about the illusory dimension of selfies emerged in our results. However, common risks related to the general consequences of selfies are considered here, even though these concerns don’t weigh as heavily among our participants as web-related anxieties. Our participants were worried about losing control of their self-images – for example, that their selfies may end up in the hands of other people who could use them for unapproved purposes; that their photos could be tampered with or retouched by others; or that their photos/identities could be stolen – especially among girls. Privacy concerns (Livingstone, 2008) tend to overshadow the positive expectations related to self-confidence and self-presentation. Indeed, self-disclosure can often result in criticism and negative opinions from others, including hostile assessments from total strangers, which explains why the adolescents in our study were well aware of the negative consequences of web-exposure. As we know, privacy disturb online self-presentation (Wang et al., 2011; Kaur et al., 2016), however, Dhir et al. (2017) recently analyzed the “privacy paradox” (Barnes, 2006), a concept that addresses privacy concerns and online self-disclosure through selfies. Privacy concerns seem to affect women more than men, and young adults more than adolescents and adults. Regardless, this doesn’t necessarily result in lower selfie activity, as privacy concerns seem to be inversely related to selfie taking/posting (Dhir et al., 2017).

The results from our sample confirm this paradox. Even though girls are more likely than boys to see selfies as a somewhat risky practice and worry about the consequences of posting selfies, this activity is more common among girls. By contrast, boys tend to see selfies (and web exposure in general) as a form of self-promotion. This is in line with Kim and Chock’s (2015) findings on the importance of popularity in shaping selfie behavior among males - a notion that was similarly confirmed in Dhir and Torsheim’s (2016) work on photo-tagging among boys. Furthermore, our study shows that the appeal of selfies among boys is also tied to ideas about excitement and sexual desire.

Our findings suggest that selfie expectations among boys and girls are quite different, and that selfie-behavior is a decidedly gendered phenomenon. As Doring et al. (2016) have noted, cultural stereotypes and social differences between boys and girls should be considered when studying the importance of selfies among adolescents and young adults.

The measure presented in this study can reliably assess adolescent expectations toward selfies and ought to be used in further research on generalized or specific selfie behavior. For instance, using selfies as both a self-promotion tool and as a means of improving one’s self-confidence needs to be considered. The tendency to show only the best part of oneself, or to present a modified representation of oneself via photos, is another aspect of selfie culture that needs to be evaluated. Moreover, if we assume that selfies can be used for self-support and aid in self-construction, then it makes sense that creating selfies in hopes of receiving the approval of others should be analyzed. Our study found that although being aware of the consequences of web-exposure encouraged a host of anxieties, it didn’t necessarily lower the frequency of selfie production among adolescents. This is probably a product of the ubiquitous nature of selfie culture nowadays, as well as the influence of one’s personality, impulsivity, emotional state, and unconscious motivations. Since identity, body-image, and related factors play significant roles in selfie behaviors, our findings point toward the necessity of promoting preventive programs that are differentiated by gender and take into account a wide array of dimensions.

### Limitations and Suggestions for Future Research

The reported findings should be interpreted by taking in account some limitations of the study.

For starters, the external validity of the findings may be limited by the sampling technique, which was based on a non-probability procedure of recruitment of the participants (see, for example, Mann, 2003; Balsamo et al., 2013). Anyway, we haven’t been able to find any other research that adequately discusses this specific topic.

Potential biases (e.g., social desirability biases) due to a self-report questionnaire are well known. However, we considered the relevant advantages provided by this kind of tool, such as: the possibility to collect a rich amount of information, the interpretability, the practicality of the administration and the participants’ motivation to share their opinions (Paulhus and Vazire, 2007).

Even though this study featured a large sample of adolescents, our research was limited to one specific geographic area. Future research should include different regions of Italy in order to compare findings from, say, Northern and Southern Italy. The findings of any study often depend on cultural aspects that should be addressed in future research. Indeed, a cross-cultural perspective could shed light on our own findings in interesting and provocative ways.

Exploratory and Confirmatory Factorial Analysis have been conducted on our sample, even though our sample was split into two half-samples. This approach was chosen due to the difficulties in tracking down a large group of participants. However, this strategy is largely adopted to validate new measures for analyzing attitudes. Generally speaking, conducting a new CFA on different samples could help us better confirm the dimensionality and validity of the measure.

The present study also has some key strengths that are worth noting. For instance, our research represents an important step in examining selfie behaviors among adolescents, providing a short and psychometrically valid measure to assess the expectations of teenagers who take part in selfie practice. Moreover, given the strong psychometrics of the instrument, researchers are encouraged to consider using this tool to assess the quality selfie-related behavior in samples of adolescents.

This study also complements previous qualitative and quantitative findings on how age and gender often shapes (and predicts) selfie behaviors (Nelson, 2013; Nguyen, 2014; Warfield, 2014; Christoforakos and Diefenbach, 2016; Dhir et al., 2017; Diefenbach and Christoforakos, 2017). It also provides a new understanding of selfie culture by engaging with a demographic that hasn’t been studied much in Italy.

Lastly, this study has some important clinical implications. Chief among them is the tendency among girls to use selfies as a means of managing various identity issues, as well as the tendency among boys to focus on sexual matters, most notably self-attractiveness issues.

## Conclusion

This study provides a new means of analyzing selfie behavior among adolescents. It examines seven important motivations and expectations that often shape the production of selfies. Our findings build on previous research on selfie behavior among millennials, while also highlighting the importance of studying the influence of age and gender on selfie-related behavior. Indeed, our selfie expectations scale should be seen as a useful tool that can help scholars and practitioners alike better understand a multifaceted and widespread phenomenon.

## Author Contributions

VB and VM both designed and conducted the study. VB led the literature search. VM analyzed the data. Both authors contribute to the interpretation and discussion of data and approved the final version of the manuscript for submission and agreed to be accountable for all aspects of the work.

## Conflict of Interest Statement

The authors declare that the research was conducted in the absence of any commercial or financial relationships that could be construed as a potential conflict of interest.
